# Sex-Specific Audience Effect in the Context of Mate Choice in Zebra Finches

**DOI:** 10.1371/journal.pone.0147130

**Published:** 2016-02-03

**Authors:** Nina Kniel, Stefanie Bender, Klaudia Witte

**Affiliations:** Research Group of Ecology and Behavioral Biology, Institute of Biology, Department of Chemistry and Biology, University of Siegen, Siegen, Germany; Claremont Colleges, UNITED STATES

## Abstract

Animals observing conspecifics during mate choice can gain additional information about potential mates. However, the presence of an observer, if detected by the observed individuals, can influence the nature of the behavior of the observed individuals, called audience effect. In zebra finches (*Taeniopygia guttata castanotis*), domesticated males show an audience effect during mate choice. However, whether male and female descendants of the wild form show an audience effect during mate choice is unknown. Therefore, we conducted an experiment where male and female focal birds could choose between two distinctive phenotypes of the opposite sex, an artificially adorned stimulus bird with a red feather on the forehead and an unadorned stimulus bird, two times consecutively, once without an audience and once with an audience bird (same sex as test bird). Males showed an audience effect when an audience male was present and spent more time with adorned and less time with unadorned females compared to when there was no audience present. The change in time spent with the respective stimulus females was positively correlated with the time that the audience male spent in front of its cage close to the focal male. Females showed no change in mate choice when an audience female was present, but their motivation to associate with both stimulus males decreased. In a control for mate-choice consistency there was no audience in either test. Here, both focal females and focal males chose consistently without a change in choosing motivation. Our results showed that there is an audience effect on mate choice in zebra finches and that the response to a same-sex audience was sex-specific.

## Introduction

Animals in a wide range of taxa are able to use public information to evaluate conspecifics [1,2,3]. By observing a signaling interaction of conspecifics, an eavesdropper or bystander, that is neither recognized by the individuals it is observing nor taking part in the interaction, can gain information about these individuals (social eavesdropping). Eavesdropping occurs when information is transmitted from one individual (sender) to another (receiver) while one or more eavesdroppers/bystanders that were not addressed pick up the signal [4]. Eavesdroppers can gather reliable information about potential mates by assessing their quality on the basis of behavioral cues, e.g. in fighting [5,6,7] or singing interactions [8,9,10,11]. Eavesdropping females gain information on the relative quality of males at little cost and/or risk [12], as evaluating potential mates might, for instance, expose them to enhanced predation risk [13] or sexual harassment [14]. By observing the mate choice of another individual, the eavesdropper can gather even more information about potential mates. Here, the eavesdropper may copy the observed decision for an individual mate or a mate of a specific phenotype, which is called mate-choice copying [15,16,17,18].

Not only does the individual that eavesdrops gain information about the two interacting individuals, but the presence of the eavesdropper may also influence the nature of the interaction if it is detected by the observed individuals. The eavesdropper is then called an audience and the change in the behavior of the observed individuals due to its presence is called ‘audience effect’ or ‘bystander effect’ [19]. The audience is not taking part in the interaction, but its presence is recognized by the interacting individuals. This audience effect has been intensively investigated in Siamese fighting fish (*Betta splendens*) (e.g., [20,21,22]) and the livebearing Poeciliidae (e.g., [7,23,24,25]), and evidence for audience effects has also been found in insects (e.g., [26,27]), mammals (e.g., [28,29,30]) and birds (e.g., [31,32,33,34,35]).

Although audience effects in the context of mate choice seem to be common, there have been relatively few studies in birds. For example, for already mated individuals, Matessi et al. [36] found that after playback of courtship display calls close to their nest, male rock sparrows (*Petronia petronia*) increased the frequency of courtship displays directed towards their mate. Baltz and Clark [32] found that male budgerigars (*Melopsittacus undulates*) limited their extra-pair courtship to times when their mates could not overserve them. Likewise, male canaries (*Serinus canaria*) reduced their extra-pair courtship in the presence of their partner [33]. Hoi and Griggio [35] found that male and female bearded reedlings (*Panurus biarmicus*) adjust their pair-bond investment mainly in response to the presence or absence of a competitor. Vignal et al. [37] found that male zebra finches altered their behavior towards their female mate depending on the mating status of conspecifics. They responded more to their partner’s voice if a mated pair was nearby. To our knowledge, there is only one study exploring audience effects in the context of mate choice in zebra finches (*Taeniopygia guttata castanotis*) so far. Dubois and Belzile [34] found that domesticated male zebra finches alter their mating preferences in the presence of a male audience (one male near each of two stimulus females) by spending more time with the formerly less preferred female. The zebra finch is a good model species to study how an audience affects mate choice, since it is a social species that lives and breeds in flocks throughout the whole year [38]. Hence, it is likely that individuals will be observed by conspecifics during mate choice. Due to the fact that both males and females highly invest in offspring, both sexes are choosy. Many studies with zebra finches have shown that both sexes choose among potential mating partners (e. g., males: [39,40]; females: [41,42,43,44]).

Why should individuals that notice that they are being observed alter their behavior? A number of explanations have been proposed for a variety of contexts (e. g., feeding: [45], social: [46], sexual: [31,32]). In promiscuous species, a rival male observing another male’s mate-choice decision might copy this choice and, therefore, be a sperm competitor to the observed male [24,25,47,48,49,50,51]. Here, the observed male might benefit from pretending a false mate choice so as to lead the rival male away from his actually preferred female. Females, when observed during mate choice by other females, might also benefit from changing their mate-choice behavior so that observing females copy the pretended mate choice, and the actually preferred male will not be sperm-depleted. Sperm-depletion could be an explanation in species in which male sperm stores are easily depleted. Evidence for audience effects in species with high rates of mating and relatively large sperm stores is lacking in at least one study [52].

Here, we investigated whether zebra finch males and females respond to an audience during mate choice. In contrast to the study by Dubois and Belzile [34] we used captive bred descendants of the wild form of the zebra finch and not domesticated ones (artificial selection), and presented only a single male audience. In contrast to other studies investigating audience effects only in one sex mate choice, we tested both sexes in the same experimental setup (but see [52]). Thus, we addressed the question whether the response to a same-sex audience is sex-specific. We also investigated whether the attention that the audience directs at the focal bird, in addition to its presence alone, would influence the behavior of the focal birds. Another contrast to the study by Dubois and Belzile [34] was that we created a new phenotype in males and females by adorning birds with a red feather on the forehead. Thereby, we were able to compare our results to a previous study on sex differences in mate-choice copying in zebra finches [18]. Kniel et al. [18] could show that female zebra finches copy the mate choice of their conspecifics for adorned males but males do not, demonstrating a sex difference in the use of public information. Furthermore, they [18] could show that both sexes have no latent preference for individuals of the opposite sex adorned with a red feather (see also: [53] for red feathers, [54] for blue feathers). However, Burley and Symanski [55] found that zebra finch females and both sexes of the monomorphic long-tailed finch (*Poephila acuticauda*) have a latent preference for conspecifics of the opposite sex with a white crest feather, while male zebra finches prefer females without crest feathers. In general, both sexes have been reported to use artificial ornamentation in their decision-making process (e. g., [18,53,54,55,56,57,58]).

Since zebra finch females copy the mate choice of other females ([18,57,58] but see [59]), one might expect them to be influenced by the presence of a same-sex individual, regarding them as some kind of competitor in mate choice. In contrast to that, males do not copy other males in mate choice [18] and might not be expected to change their preferences. However, Dubois & Belzile [34] found an audience effect in domesticated male zebra finches. Moreover, since the zebra finch is a socially monogamous species, in which pairs stay together for more than one breeding period, access to potential mates is limited. Therefore, changing their mate choice might expose both sexes to the risk of having to mate with a partner of low quality, or even remain unpaired. Hence, there are arguments for and against finding an audience effect in zebra finches.

## Methods

### Study Species

Birds used in this study were sexually mature, captive bred, F_8-11_ descendants (females: mean age about 31 months, minimum: 8 months, maximum: 46 months; males: mean age about 30 months, minimum: 8 months, maximum: 44 months) of wild zebra finches that were exported from Northern Victoria, Australia in 1992. They were kept in six aviaries (four aviaries: 2 x 1.65 x 2.30 m³, two aviaries: 2.25 x 1.05 x 2.30 m³), separated by sex after maturation for at least six months before the experiments. Aviaries were separated visually from each other, but birds could communicate acoustically. The air-conditioned room (6.80 x 4 x 2.40 m³) (Temperature: 24° ± 1°C, Humidity: 60% ± 1%) had windows at two sides, allowing sunlight to enter the room and was additionally illuminated with fluorescent lighting including UV-range at a 14:10 h light:dark photoperiod. Both sexes wore numbered orange or white leg bands, or silver metal leg bands (neutral in mate choice: [56,60,61]). Each aviary contained several branches, coconut fibers as nest building material, eight nest boxes as well as sand, food and water (for drinking and bathing) *ad libitum*. Zebra finches were fed daily with food consisting of a mixture of seeds containing Senegal, red, yellow and Canary millets, sprouted birdseed, cucumber, chickweed and crushed eggshells.

### Experiments

**Fig 1:**
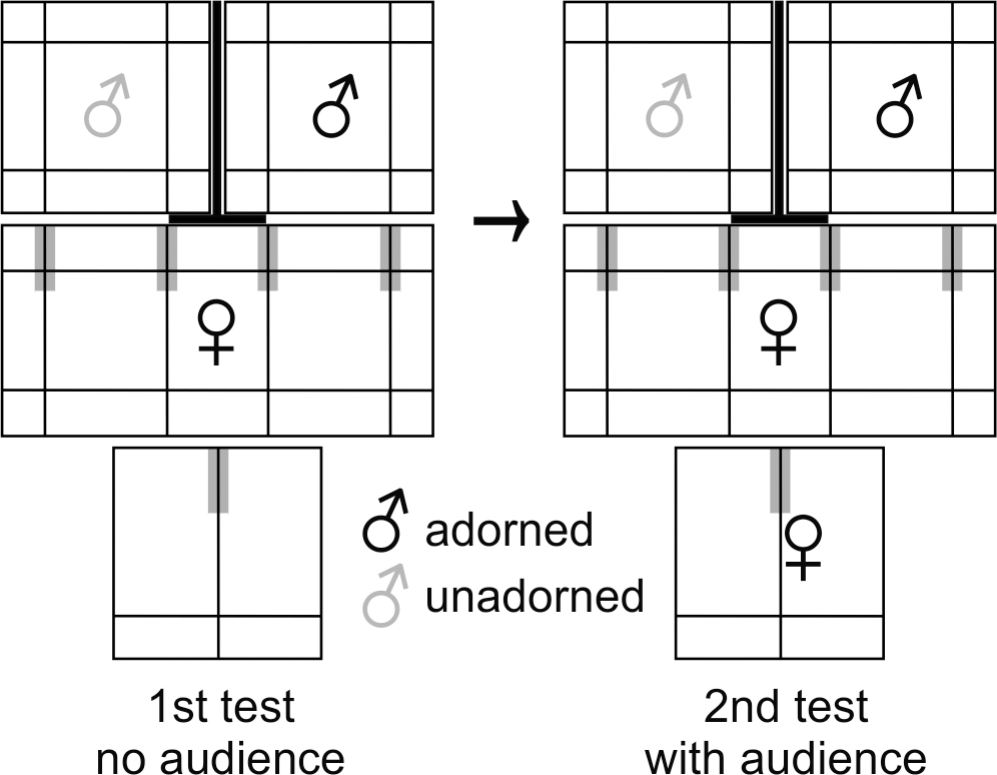
Experimental setup. Top-view onto the experimental setup for female and male mate-choice tests (Fig shows female mate-choice test). Lines within the cages are perches. Bold lines are screens. Gray zones are mate-choice zones. 1st test no audience = first mate-choice test without an audience. 2nd test with audience = second mate-choice test with an audience.

Experiments were conducted between June and December 2012 in an air-conditioned room (2.20 x 2.10 x 2.40 m^3^) without windows (Temperature: 24° ± 1°C, Humidity: 60% ± 1%) and a 14:10 h light:dark photoperiod with fluorescent lighting including UV-range. Cages of stimulus birds (each 49 x 43 x 50 cm^3^) were placed side by side, the cage of the focal bird (97 x 43 x 52 cm^3^) was placed in front of them. The audience bird’s cage (49 x 43 x 50 cm^3^) was located on the other side of the focal bird’s cage (Fig 1). Each cage contained water, food and sand *ad libitum* in little bowls on the ground. Stimulus birds’ cages had four perches: one low perch parallel and close to the front (10 cm above the bottom of the cage), one high perch parallel and close to the backside (35 cm) and two additional perches parallel to the side of the cage in middle height (20 cm). The cage of the focal bird was equipped like the stimulus birds’ cages and had four perches of choice in middle height (Fig 1). The audience bird’s cage was only equipped with one perch in middle height (20 cm) parallel to the cage sides and in the middle of the cage, and a high perch in the back (35 cm), parallel to the cage front (Fig 1). A screen, placed between the stimulus birds’ cages, prevented visual contact between the two stimulus birds. A paper screen (18 cm wide and 49 cm high), located on the front and in the middle of the focal bird’s cage, prevented the focal bird from seeing both stimulus birds at the same time when being in direct proximity of one of the stimulus birds’ cages.

All birds were kept in their cages in the room where the experiments were performed for at least 15 hours before we started the test the next morning, to acclimate before testing, in visual but not acoustic isolation from other birds via additional screens. The cages for the first mate-choice test were placed on a table as described above. The other cages were placed in a shelf and exchanged during the test the next day where necessary.

**Fig 2:**
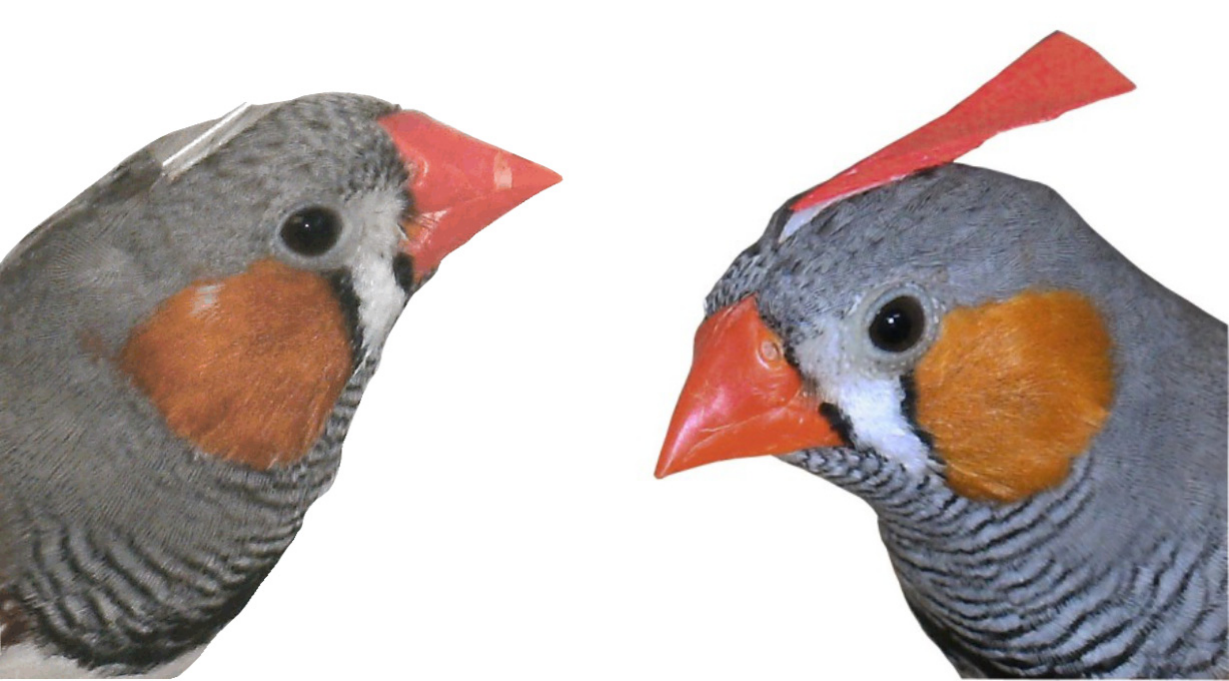
Adornment. Pictures of an unadorned stimulus male (left) and an adorned stimulus male (right). Stimulus females were adorned in the same way.

To create two distinct phenotypes (Fig 2), stimulus birds (but not the audience) were either adorned with a red feather, standing upright like a crest and representing a conspicuous trait, or equipped with a piece of a gray flight feather (unadorned), representing the common phenotype, when they were caught. Red feathers were cut out of a red feather boa along the quill (length: 2 cm, width: 4–5 mm) and were elliptically shaped. Flight feathers (collected from moulting birds from aviaries) that matched the color of the forehead feathers were cut to triangles (maximum edge length 5 mm). Both were glued to the forehead onto the natural feathers of the birds with double-sided tape; pieces of flight feathers were completely glued onto the forehead feathers, the red feather was glued onto the forehead feathers with its base (the base was about the same size as the piece of flight feather), so that the rest of the feather protruded from the back of the head (Fig 2). This way, all stimulus birds were handled equally, but differed in their visible adornment. This method was successfully used before in a number of experiments with zebra finches [18,53,54] and the Javanese mannikin (*Lonchura leucogastroides*) [62,63,64].

### General Procedure

Each test consisted of two parts. In the first part (first mate-choice test) focal birds could choose between an adorned (red feather) and an unadorned stimulus bird of the opposite sex (mate choice without an audience). In the second part (second mate-choice test) focal birds could again choose between the same two stimulus birds while an audience bird (same sex as focal bird, not adorned) was watching. Since we introduced a novel phenotype in our experiments, we first tested whether focal birds had a latent preference for this trait in the absence of an audience bird. Other studies found no latent preference for the red feather [18,53], and Kniel et al. [18] found female birds to prefer unadorned males in one control. In order to be able to find an effect of an audience on mate choice for a new trait, the baseline of the preferences of the focal birds has to be tested first. Therefore, rather than using a mixed design (i.e. testing half of the focal birds in a reversed order: first with an audience and second without an audience), we additionally conducted a control for mate-choice consistency.

### Audience Experiments

By removing the screen that prevented the focal bird from seeing the stimulus birds, we started the first mate-choice test, which lasted 40 min (2 x 20 min). After 20 min we switched the position of stimulus birds’ cages to control for side biases by first entering the screens again, then switching the position of the stimulus birds’ cages and giving the birds up to five minutes before removing the screens again, which is sufficient for birds to calm down [18,53,54]. This test duration was successfully used for mate-choice experiments in zebra finches [18,53,54]. We measured the attractiveness of stimulus birds as the time (s) the focal bird spent perching on the outer one-third of the perches of choice adjacent to the stimulus birds’ cages (mate-choice zone; gray area in Fig 1). We did not include time spent in front of stimulus birds below the perches, because it was not clear whether the focal bird was interested in the stimulus birds or in the food in the stimulus birds’ cages. These and all other positions were scored as no-choice positions. We recorded the position and behavior of the focal bird every 10 s. If the focal bird changed position during the 10 s-interval, 5 s were scored, otherwise 10 s. This method is an established measurement to determine sexual preferences in zebra finches [18,53,54,65]. Thereby, we measured the time the focal bird spent with the respective stimulus birds and we also calculated their choosing motivation (total time spent in both mate-choice zones during the 2 x 20 min mate-choice test). Additionally, we measured song frequency of focal, stimulus and audience males (whether or not males sang within a 10 s-interval, either directed at their conspecifics or undirected). Male song rate is known to influence female mate choice as they spent more time with males that sing more often compared to those that sing less often [43].

After this first mate-choice test we again positioned the screens to prevent visual contact between focal and stimulus birds and exchanged the empty audience cage with a new cage containing the audience bird (same sex as focal bird). We conducted the second mate-choice test in the same way as the first mate-choice test, which also lasted for 2 x 20 min, after a 5 minute pause for the focal bird to become accustomed to the presence of the audience bird. Here, we quantified the time the audience bird spent close to the focal bird (within the outer one-third close to the focal bird’s cage of his/her perch in middle height; Fig 1) and counted how often the audience male sang as described above.

Focal birds that showed a side bias, that means they spent more than 80% of their choosing time on the same side of their cage during the first mate-choice test, even though we had switched the position of the stimulus birds’ cages, were excluded from the analysis in accordance to other studies [18,66,67,68,69]. We tested a total number of 23 males and 20 females in the audience experiment.

After each test we measured the body weight of all birds and placed them back into their aviaries or cages. We used each focal bird only once as a focal bird, but later on up to twice as an audience bird. Stimulus birds were used in up to two tests, but always in different combinations and both as an adorned and an unadorned stimulus bird. Audience birds were also used in up to two tests, but never afterwards as focal birds. We reused birds because the number of birds available for experimentation was limited. Stimulus and audience birds were not known to focal birds and were taken from different aviaries as focal birds. We balanced ages of all birds by mixing focal, stimulus and audience birds so that all age combinations were used. We also balanced the use of sexually experienced and inexperienced birds. By this, we made sure that no age effect or effect of sexual experience could be responsible for the outcome of the experiments.

Throughout the whole testing time (10 min before starting the first mate-choice test until the last mate-choice test was completed) we played zebra finch sounds (recorded in the aviary-room January 2008) through a loudspeaker (Speed Link, Brave 2.0 Stereo Sound System) as done in Kniel et al. [18]. Since zebra finches live in flocks, they tend to be relatively inactive if they do not hear calls of conspecifics.

### Control

The control was designed to test for mate-choice consistency. The control was identical to the experimental procedure, but in the second mate-choice test, the cage for the audience bird was empty, therefore no audience bird was watching the focal bird during the second mate-choice test. This control was performed for both sexes equally. We tested a total number of 21 focal males and 19 focal females, none of which were used as test birds before.

### Ethics statement

All behavioral experiments were performed under the permission of the County Veterinary Office, Siegen, Germany (permit numbers: 53.6 55–05). We declare that this study was carried out in strict accordance with the recommendations in the Guide for the Care and Use of Laboratory Animals of the German Right of Animal Welfare (Tierschutzgesetz).

### Statistical Analysis

We analyzed the overall choosing motivation as the proportion of total time spent in both mate-choice zones and compared them between the two mate-choice tests with a Wilcoxon matched-pairs test. We analyzed the time focal birds spent within the mate-choice zones in front of the stimulus birds as a measure of mate choice. To test whether focal birds showed a preference for one of the two phenotypes, we tested the mate-choice scores for adorned males (time spent with the adorned stimulus / time spent with both the adorned and the unadorned stimulus) against a 50% expectation using a one-sample t-test. We used mate-choice scores for the adorned stimulus bird to test whether these scores changed from the first to the second part of a test with an audience present, but was unchanged in the control runs. We transformed mate-choice scores arcsine-square-root to obtain normally distributed data and performed a repeated-measures ANOVA (RM-ANOVA with ‘mate-choice tests’ as within-subject factor). Where ANOVA results did not conform to the assumption of sphericity, Greenhouse-Geisser approximations were used. If ANOVA results indicated an effect, we performed a paired t-test to specify how time spent with the stimulus birds changed. Additionally, we wanted to test whether the behavior of the audience birds, instead of their presence alone, might influence the mate choice of focal birds. Therefore, we tested whether there is a correlation (Pearson correlation) between the time the audience bird spent in the front of its cage close to the focal bird and the change in time spent of the focal bird with the respective stimulus birds expressed as a log-ratio difference (log (score adorned first test/score unadorned first test)–log (score adorned second test/score unadorned second test)). Finally, we statistically compared weight of birds (given are in parentheses the median and 1st and 3rd quartile [g]) and number of intervals with song (directed and undirected) using Wilcoxon matched-pairs tests, Mann-Whitney-U tests, and paired and unpaired t-tests. Statistical analyses were performed using SPSS (IBM SPSS Statistics 22). Significance levels were set at α = 0.05. All p values are two-tailed. Data are available as an online file (S1 Table).

## Results

### Audience Experiment in Males

**Fig 3:**
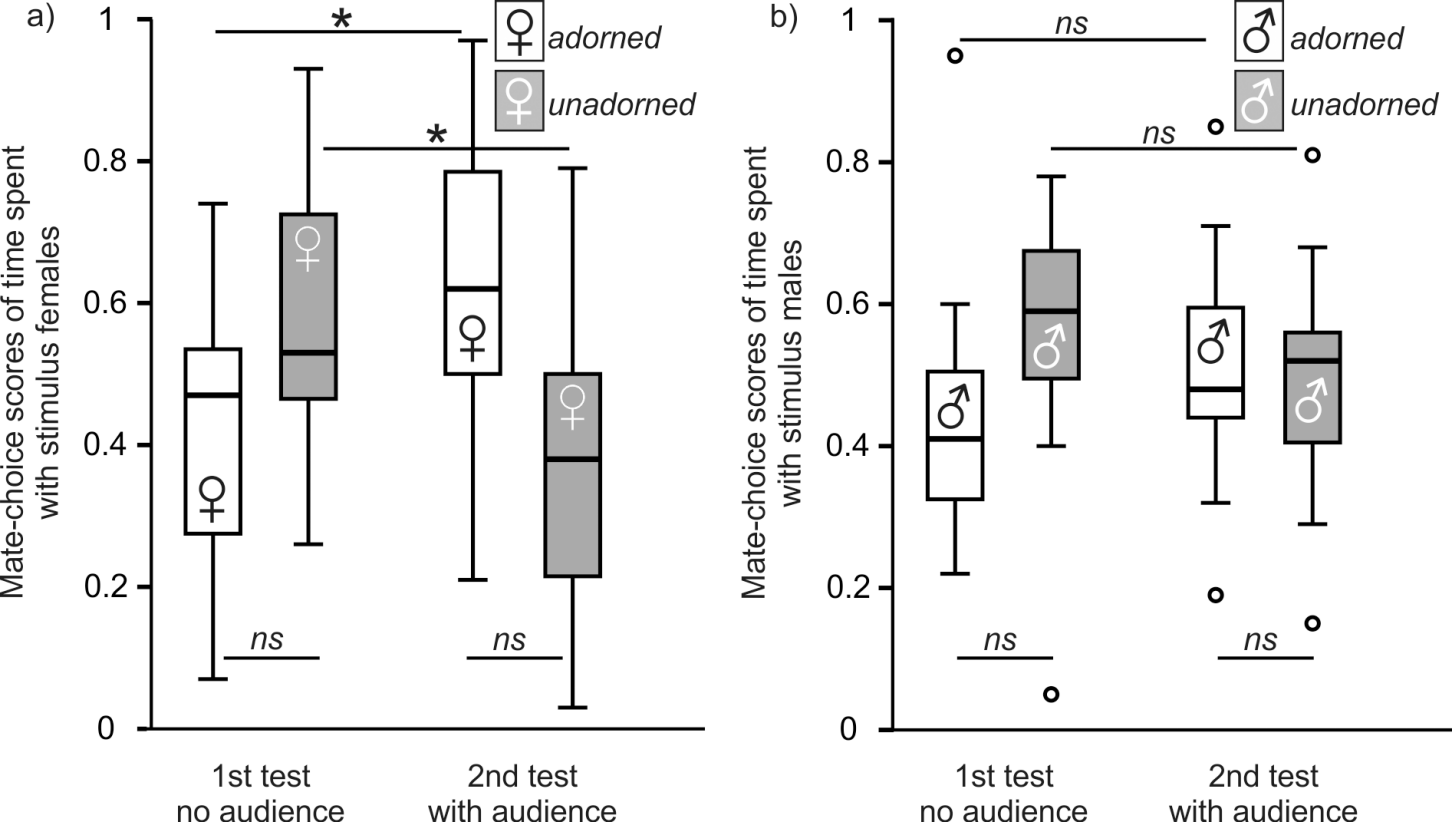
Audience Experiments in males (a) and females (b). Box plot showing median, 1st and 3rd quartile, 95% confidence limits and open points as outliers for mate-choice scores of time spent with stimulus birds. 1st test = first mate-choice test (no audience), 2nd test = mate-choice test (audience). ns = not significant. * = p < 0.05.

**Fig 4:**
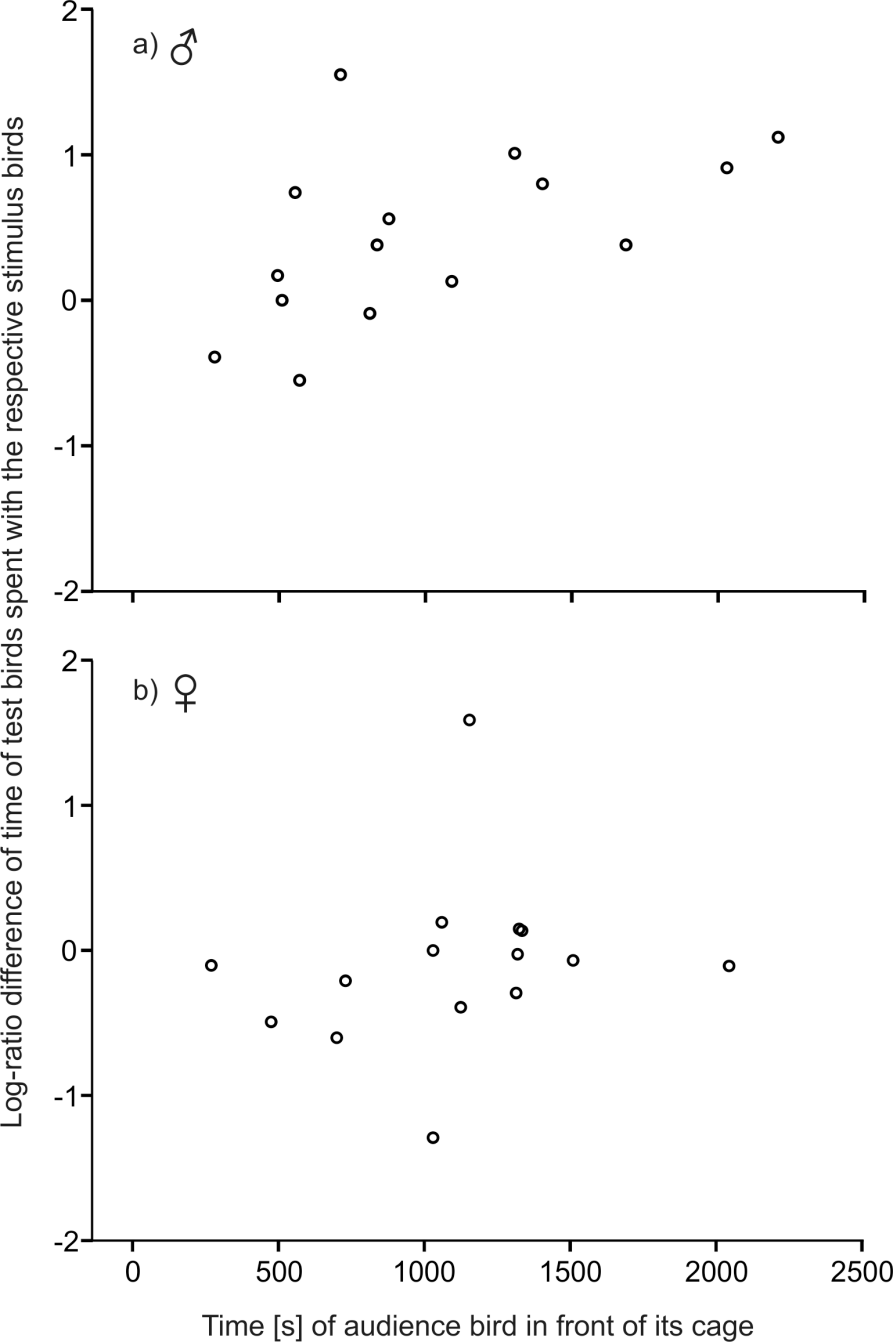
Correlation. Correlation between the time the audience bird spent in the front of its cage close to the focal males (a) or focal females (b) and the change in time spent of focal birds with the respective stimulus birds expressed as a log-ratio difference.

Seven of the 23 males were excluded from the audience experiment because they showed a side bias. One additional male was excluded because the adorned female lost her red feather during the test. Overall choosing motivation (total time spent with stimulus birds) was not significantly different between the first and the second mate-choice test (Wilcoxon matched-pairs test: Z = -1.250, n = 15, p = 0.211). Mate-choice score of time spent with adorned females was affected by mate-choice test (RM-ANOVA: mate-choice tests: F_1,14_ = 8.977, p = 0.010; Fig 3A). Focal males showed a tendency to spend more time with adorned females in the second (one-sample t-tests: t = 2.085, df = 14, p = 0.056), but not in the first mate-choice test (one-sample t-tests: t = -1.598, df = 14, p = 0.132). Time spent with adorned stimulus females increased significantly from the first to the second mate-choice test (paired t-test: t_14_ = -2.996, p = 0.040), whereas time spent with unadorned females decreased significantly (paired t-test: t_14_ = 2.993, p = 0.030). The change in time spent with the respective stimulus females was positively correlated with the time that the audience male spent in front of its cage close to the focal male (Pearson correlation: r = 0.535, n = 15, p = 0.040; Fig 4A). Focal males did not sing undirected in more intervals during the first than during the second mate-choice test (paired t-test: t_14_ = 1.782, p = 0.096). They did not direct more song at adorned than at unadorned females during both mate-choice tests (Wilcoxon matched-pairs tests: first test: Z = -0.120, p = 0.908; second test: Z = 0.647, p = 0.518). However, they directed song less often at both females during the second than during the first mate-choice test (Wilcoxon matched-pairs tests: adorned: Z = -2.198, p = 0.028; unadorned: Z = -2.414, p = 0.016). Audience males directed their song at focal males in four of the 15 tests, whereas focal males directed their song at audience males in only two tests. Focal males (median: 11.75 g, 1st quartile: 11.40 g, 3rd quartile: 12.90 g) were of similar weight as audience males (median: 11.33 g, 1st quartile: 11.12 g, 3rd quartile: 12.40 g) (unpaired t-test: t_28_ = 0.964, p = 0.343). Adorned females (median: 12.00 g, 1st quartile: 11.60 g, 3rd quartile: 12.51 g) were of similar weight as unadorned females (median: 12.27 g, 1st quartile: 11.89 g, 3rd quartile: 12.55 g) (unpaired t-test: t_28_ = -0.239, p = 0.813).

### Audience Experiment in Females

Five of the 20 tested females were excluded from our analysis because they showed side biases. Overall choosing motivation declined significantly from the first to the second mate-choice test (Wilcoxon matched-pairs test: Z = -2.613, n = 15, p = 0.009). Time spent with adorned stimulus males was not affected by mate-choice test (RM-ANOVA: mate-choice tests: F_1,14_ = 0.033, p = 0.403; Fig 3B). Time spent with the respective stimulus males in both mate-choice tests did not differ from a 50% expectation (one-sample t-tests: first test: t_14_ = -1.110, p = 0.286; second test: t_14_ = 0.177, p = 0.826). The change in time spent with the respective stimulus males was not correlated with the time that the audience female spent in front of its cage close to the focal female (Pearson correlation: r = 0.2, n = 15, p = 0.474; Fig 4B). Therefore, the time that the audience female spent close to the focal female did not influence the mate choice of focal females for those males. The number of intervals with undirected song did not differ between stimulus males (Mann-Whitney-U-tests: first test: Z = -0.240, p = 0.810; second test: Z = -0.364, p = 0.716) and mate-choice tests (Wilcoxon matched-pairs tests: adorned: Z = -0.524, p = 0.600; unadorned: Z = -1.367, p = 0.172). Adorned and unadorned stimulus males did not differ in number of intervals with song directed at focal females during both mate-choice tests (unpaired t-tests: first test: t_28_ = 0.032, p = 0.975; second test: t_28_ = -1.344, p = 0.190). Adorned stimulus males (paired t-test: t_14_ = 2.860, p = 0.013) but not unadorned stimulus males (paired t-test: t_14_ = 1.776, p = 0.098) sang less often directed at focal females during the second than during the first mate-choice test. Focal females (median: 12.47 g, 1st quartile: 11.48 g, 3rd quartile: 12.99 g) were of similar weight as audience females (median: 12.31 g, 1st quartile: 11.28 g, 3rd quartile: 12.62 g) (unpaired t-test: t_28_ = 0.131, p = 0.897). Adorned males (median: 11.78 g, 1st quartile: 11.31 g, 3rd quartile: 12.22 g) were of similar weight as unadorned males (median: 11.79 g, 1st quartile: 11.13 g, 3rd quartile: 12.29 g) (unpaired t-test: t_28_ = -0.209, p = 0.836).

### Male control

**Fig 5:**
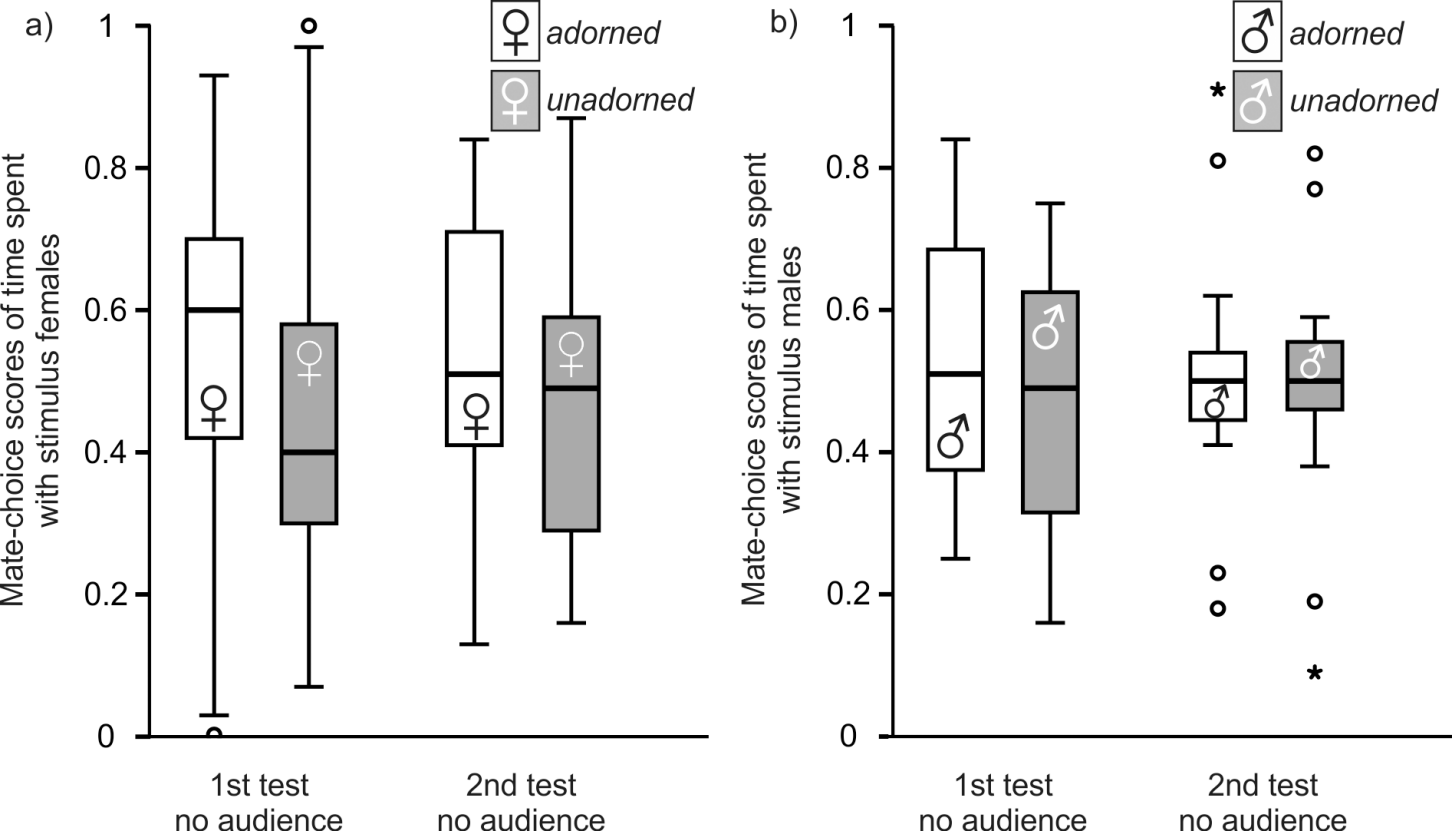
Male (a) and female (b) control. Box plot showing median, 1st and 3rd quartile, 95% confidence limits and open points as outliers and stars as extremes for mate-choice scores of time spent with stimulus birds. 1st test = first mate-choice test (no audience), 2nd test = mate-choice test (no audience).

Six of the 21 tested males were excluded from this control because of side biases. Overall choosing motivation was not significantly different between the first and the second mate-choice test (Wilcoxon matched-pairs test: Z = -1.505, n = 15, p = 0.132). Time spent with adorned stimulus females was not affected by mate-choice test (RM-ANOVA: mate-choice tests: F_1,14_ = 0.017, p = 0.899; Fig 5A). Time spent with the respective females in both mate-choice tests did not differ from a 50% expectation (one-sample t-tests: first test: t_14_ = 0.344, p = 0.736; second test: t_14_ = 0.284, p = 0.780). Focal males did not sing undirected in more intervals during the first than during the second mate-choice test (Wilcoxon matched-pairs test: Z = -1.339, n = 15, p = 0.180). They did not direct more song at adorned than at unadorned stimulus females during both mate-choice tests (Wilcoxon matched-pairs tests: first test: Z = -0.677, p = 0.498; second test: Z = -0.647, p = 0.518). They sang less often directed at adorned stimulus females (Wilcoxon matched-pairs test: Z = -2.043, n = 15, p = 0.041) but not at unadorned stimulus females (Wilcoxon matched-pairs test: Z = -1.706, n = 15, p = 0.088) during the second than during the first mate-choice test. Focal males weighed a median of 11.58 g (1st quartile: 10.76 g, 3rd quartile: 12.77 g). Adorned stimulus females (median: 12.07 g, 1st quartile: 11.21 g, 3rd quartile: 12.25 g) were of similar weight as unadorned females (3rd quartile: 12.24 g, 1st quartile: 11.21 g, 3rd quartile: 12.83 g) (unpaired t-test: t_28_ = -0.785, p = 0.439).

### Female control

We excluded four of the 19 tested females from this control because of side biases. Overall choosing motivation was not significantly different between the first and the second mate-choice test (Wilcoxon matched-pairs test: Z = -0.738, n = 15, p = 0.460). Time spent with adorned stimulus males was not affected by mate-choice test (RM-ANOVA: mate-choice tests: F_1,14_ = 0.150, p = 0.704; Fig 5B). Time spent with the respective males in both mate-choice tests did not differ from a 50% expectation (one-sample t-tests: first test: t_14_ = 0.492, p = 0.630; second test: *t*_14_ = 0.201, p = 0.843). The number of intervals with undirected song did not differ between stimulus males (Mann-Whitney-U-tests: first test: Z = -0.099, p = 0.921; second test: Z = -0.025, p = 0.980) and mate-choice tests (Wilcoxon matched-pairs tests: adorned: Z = -0.634, p = 0.526; unadorned: Z = -1.510, p = 0.131). Adorned and unadorned stimulus males did not differ in number of intervals with song directed at focal females during both mate-choice tests (paired t-tests: first test: t_28_ = 1.111, p = 0.276; second test: t_28_ = 0.588, p = 0.561). Adorned and unadorned stimulus males sang less often directed at focal females during the second than during the first mate-choice test (paired t-tests: adorned: t_14_ = 3.494, p = 0.004; unadorned: t_14_ = 2.296, p = 0.038). Focal females weighed a median of 12.44 g (1st quartile: 11.57 g, 3rd quartile: 12.82 g). Adorned males (median: 11.96 g, 1st quartile: 11.06 g, 3rd quartile: 12.63 g) were of similar weight as unadorned males (median: 11.41 g, 1st quartile: 10.83 g, 3rd quartile: 12.23 g) (unpaired t-test: t_28_ = 1.052, p = 0.302).

## Discussion

In the present study, we investigated whether male and/or female zebra finches show an audience effect in the context of mate choice between two different phenotypes in the opposite sex. Focal male zebra finches changed their mate choice when a same-sex audience bird was present. They significantly increased the time spent with adorned stimulus females between the first (without audience) and the second mate-choice test (with audience), whereas they decreased the time spent with unadorned stimulus females. In contrast to that, focal females did not change their mate choice in the presence of an audience female. However, focal females spent less time with both stimulus males (choosing motivation) in the presence of the female audience, whereas focal females in the control did not change their choosing motivation. Neither sex showed a latent preference or rejected the adorned phenotype during the first mate-choice test, which is consistent with previous experiments (red feather: [18,53], blue feather: [54]).

Since male zebra finches changed their mate choice when a same-sex audience was present, they tended to prefer adorned females over unadorned females in the second mate-choice test. Since they chose consistently in our controls, this change can only be explained by the presence of a male audience during the second mate-choice test in the audience experiment. This result is consistent with a study by Dubois and Belzile [34] with domesticated zebra finch males, where, in the presence of an audience (two males), males spent more time with the female that was considered the less attractive one (with whom the males spent less time) in the control with no audience. In general, the two situations in our experiment and in that by Dubois and Belzile have been different because we used one audience male while they used two audience males. In their situation, test males were faced with a higher possibility of remaining unpaired if both audience males would succeed in courting the respective females compared to our experiment in which only one audience male was present. The experiment by Dubois and Belzile, therefore, simulated a higher pressure for test males than our experiment. Our results demonstrate, that even under less pressure, zebra finch males show an audience effect in the presence of a potential rival.

Additionally, we found that the change in male mate choice was positively correlated with the time the audience male spent close to focal males. The more time the audience males spent in the front of their cage close to the focal males, the greater was the change in mate choice (spending more time with adorned stimulus females and less time with unadorned stimulus females) of focal males. Obviously, males recognized the presence of an audience male and regarded him as a potential rival, and responded accordingly by altering the extent to which they changed their choosing behavior. Despite the small sample size, our results show that not only the presence of the audience, but also the behavior of the audience (attention directed at the focal male and the stimulus females) may influence male zebra finches in their mate choice. Here, further experiments are needed to specify what kind of behavior of the audience exactly influences the change in male mate-choice decisions.

Why do males change their mate choice behavior in the presence of an audience male? In promiscuous species sperm competition is a likely explanation for the change in male behavior (e.g., [24,47,48,49,50,51,70]); however, this explanation is unlikely in this socially monogamous species. Extra-pair copulations may occur, but they are generally rare in zebra finches [71,72,73]. In an earlier study Kniel et al. [18] have shown that male zebra finches do not copy the mate choice of other males. Thus, pretending a preference to lead males away from a potential mate would make no sense. Another explanation for the audience effect in zebra finch males might be that the presence of an audience simply diverted the attention of males and led to the change in mate choice [70]. This phenomenon (‘split-attention hypothesis’) suggests that the presence of an audience generally renders accurate mate choice a difficult task. If this was the case, then a social living bird like the zebra finch would generally not be able to adequately choose mates, which is highly unlikely. As Dubois and Belzile [34] proposed, zebra finch males might change their preference with respect to their ‘mate-getting ability’ to reduce the risk of being rejected by their preferred mate and remaining unpaired. If the audience male had a higher chance to pair with the female than the focal male, or if focal males have no means of knowing their chances, then focal males should change their preference and invest more in courting the other female.

Males sang less often directed at both females during the second (with audience) than during the first mate-choice test (without audience). However, this decrease cannot simply be explained by the presence of the audience, since males also sang less often directed at adorned females during the second than during the first mate-choice test in controls (without an audience). This decrease in directed singing activity might arise because a physical interaction was prevented due to the experimental setup and males simply decreased their effort due to the limited response by females.

Females changed their mate-choice behavior in the presence of an audience as well, but in a different way from males. Females did not alter their mate choice in the presence of a female audience. However, the total time females spent with both stimulus males decreased when the audience female was present, and focal females mostly spent their time in the middle of the cage, which explains the decrease in choosing motivation. In controls, females also showed no change in their preference, but choosing motivation did not differ between the first and the second mate-choice test. A decrease in choosing motivation in our experiments can, therefore, not be explained by a general loss of interest over time. Hence, the presence of the audience female changed the focal female’s behavior, i.e. overall time spent with stimulus birds, but not their preference. The zebra finch is a highly social species and females like to associate with other females [36]. The decrease in choosing motivation might represent a conflict between spending time with potential mates and spending time with conspecific females as social companions. However, this is not very likely since females did not spend that lost time with audience females, but rather in the middle of the cage. As a socially monogamous species and opportunistic breeder, females are under high pressure to find the best possible mate. Although the zebra finch engages in biparental brood care, females are still assumed to invest more in offspring due to the higher costs of egg production, which makes it evident that females should be choosy when engaging with a male. This is underlined by a study of Holveck and Riebel [74], that showed that females of low quality (manipulated by rearing conditions) preferred males of low quality over males of high quality. When paired, quality-matched pairs had a shorter latency to lay eggs, indicating that quality-matched birds were accepted faster as partners. The presence of another female, as will most likely be the case in nature, should, therefore, not influence females’ mate choice.

In contrast to males, we found no correlation between the change in female mate choice, time spent with the respective stimulus males, and the time the audience females spent close to focal females. It did not matter how much attention the audience female directed at the focal female and the stimulus males, further underlining that the presence of a female audience does not influence zebra finch female mate choice.

Since female zebra finches are known to copy the mate choice of their conspecifics [18,57,58], one might think that pretending a preference for one of the two stimulus males might lead rival females away from the other male. Experiments have demonstrated that zebra finch females will copy the choice for an individual male only after an observation period of two weeks [57], whereas they will copy the choice for a phenotype (generalize between males) after a relatively short time period of two hours [18]. Therefore, observing females do not seem to represent an immediate threat to the observed female. Such a misleading behavior would, therefore, not be adaptive for female zebra finches at first sight. However, the decrease in choosing motivation could still be a reaction to the audience in the sense that females spent less time exhibiting their mate choice so as not to make it too clear which male is their preference. Although rates of extra-pair copulations are low [71,72,73] there is a possibility that at least some observing/copying females could be sperm competitors to focal females in this context [75]. Since male zebra finches have relatively small testes [76] and small sperm supplies in their seminal glomeruli [77], males engaging in extra-pair copulation might not be able to fertilize eggs for some time, which could lead to a delayed breeding cycle for the focal female or unfertilized eggs in their clutch.

Our experiments with captive bred descendants of the wild form of the zebra finch thus replicate the results of Dubois and Belzile [34] where males of the domesticated form showed an audience effect. We not only replicated the study by Dubois and Belzile, we also tested females in the same design. We showed that female zebra finches of the wild form, in contrast to males, show an audience effect as a reduction of choosing motivation. We could not find differences between the stimulus birds in other measurements (weight, song of stimulus males) between the respective stimulus birds. Therefore, we believe that we presented comparable stimuli that only differed in their ornamentation. Thus, we found that the response to a same-sex audience in the context of mate choice in zebra finches is sex-specific.

## Supporting Information

S1 Table Supporting information. (XLSX)
